# HIV-1 clade C escapes broadly neutralizing autologous antibodies with N332 glycan specificity by distinct mechanisms

**DOI:** 10.1186/s12977-016-0297-2

**Published:** 2016-08-30

**Authors:** Suprit Deshpande, Shilpa Patil, Rajesh Kumar, Tandile Hermanus, Kailapuri G. Murugavel, Aylur K. Srikrishnan, Suniti Solomon, Lynn Morris, Jayanta Bhattacharya

**Affiliations:** 1 HIV Vaccine Translational Research Laboratory, NCR Biotech Science Cluster, Translational Health Science and Technology Institute, Faridabad, Haryana India; 2 YRG Care Center for AIDS Research & Education, Chennai, 600113 India; 3 National Institute of Communicable Diseases, Johannesburg, South Africa; 4 International AIDS Vaccine Initiative, New York, NY USA

**Keywords:** HIV-1, Neutralizing antibody, Envelope, Plasma

## Abstract

The glycan supersite centered on N332 in the V3 base of the HIV-1 envelope (Env) is a target for broadly neutralizing antibodies (bnAbs) such as PGT121 and PGT128. In this study, we examined the basis of resistance of HIV-1 clade C Envs obtained from broadly cross neutralizing (BCN) plasma of an Indian donor with N332 specificity. Pseudotyped viruses expressing autologous *envs* were found to be resistant to autologous BCN plasma as well as to PGT121 and PGT128 mAbs despite the majority of Envs containing an intact N332 residue. While resistance of one of the Envs to neutralization by autologous plasma antibodies with shorter V1 loop length was found to be correlated with a N332S mutation, resistance to neutralization of rest of the Envs was found to be associated with longer V1 loop length and acquisition of protective N-glycans. In summary, we show evidence of escape of circulating HIV-1 clade C in an individual from autologous BCN antibodies by three distinct mechanisms.

The HIV-1 envelope (Env) protein that mediates entry of viral RNA into the cellular cytoplasm is the target of neutralizing antibodies. During the course of the infection, HIV-1 evolves within an individual to escape the humoral immune pressure by selection of mutations, alterations of the glycosylation pattern and varying loop lengths. Variation in lengths and glycosylation patterns in the hypervariable loops of viral Env, particularly in the V1V2 loop has been shown to be associated with alterations in virus entry and neutralization [1–14].

Neutralizing antibodies to autologous circulating HIV-1 develop in most infected individuals within 6 months [5, 15, 16], however only in some individuals, neutralizing antibodies with considerable breadth and potency develops over time [17, 18]. Broadly neutralizing monoclonal antibodies (bnAbs) isolated from individuals infected with HIV-1 have identified major targets in the CD4 binding site (CD4bs), the membrane proximal external region (MPER), the trimer apex (V1/V2), the V3-glycan supersite and the gp120/gp41 interface on the HIV-1 Env [19]. Variable loop length (particularly the V1V2 loop) and glycosylation signatures within these loops have been demonstrated to be selectively associated with resistance and enhanced sensitivity to some bnAbs [6, 7, 9, 10, 20–24].

Glycan supersites in the V3 region of HIV-1 envelope form vulnerable targets that are exploited by potent and broadly neutralizing monoclonal antibodies (bnAbs) such as PGT121 and PGT128 [25]. The glycan N332 residue in the V3 base has been demonstrated to represent an important supersite of vulnerability for comprehensive antibody mediated virus neutralization and is currently aiding design and development of an effective vaccine. In the case of mAbs PGT121 and PGT128 that target the V3-glycan supersite, loss of the glycan at position 332 is often associated with resistance [26]. Recently, Goo et al. and Sok et al. [26, 27] reported that some viruses despite the presence of key N301 and N332 V3 glycans were found to be resistant to the potent and broadly neutralizing mAbs, PGT121 and PGT128. They suggested that altered conformation of Env due to unknown mechanisms resulted in neutralization resistance of viruses to these mAbs. Recently, van den Kerkhof et al. [14] showed association of elongated V1 loop length with resistance to patient derived primary Envs to PGT135 mAb. In the present study, we examined the basis of resistance of HIV-1 clade C Envs to contemporaneous BCN plasma (INDO-SA 2007) obtained from a slow progressing Indian patient whose specificity mapped to the N332 at the V3 base. By examining HIV-1 Envs obtained from BCN plasma of an Indian patient, we found that longer V1 loop length hinders the bnAbs such as PGT121 and PGT128 to access the N332 glycan epitope. Our observation provides information that explains the basis of resistance of HIV-1 variants that are naturally resistant to bnAbs targeting N332 glycan epitope.

We screened 100 HIV-1 positive plasma samples obtained from antiretroviral therapy (ART) naïve slow progressing Indian donors with a CD4 count >350 mm^3^ for the presence of broadly neutralizing antibodies to HIV-1 clade C viruses from India (n = 9) and South Africa (n = 8). Of the 21 plasma samples that were found to cross neutralize >50 % of the pseudoviruses at 1:100 dilution, we identified an Indian donor (INDO-SA 2007) whose plasma showed the maximum breadth and potency with median ID50 of 770 (Fig. 1a). The INDO-SA 2007 plasma showed geometric mean titer of >749 when tested against a larger panel of 28 HIV-1 Env pseudotyped viruses primarily comprising Envs of Indian and South African clade C origins (Table 1). Interestingly, we found that majority of the panel Envs examined contains N332, which is an important target of neutralizing antibodies such as PGT128 [11]; however the INDO-SA 2007 BCN plasma also neutralized Envs that lacks N332 but contains N334 which has been demonstrated to compensate the function for N332 in a context dependent manner [28].

**Fig. 1:**
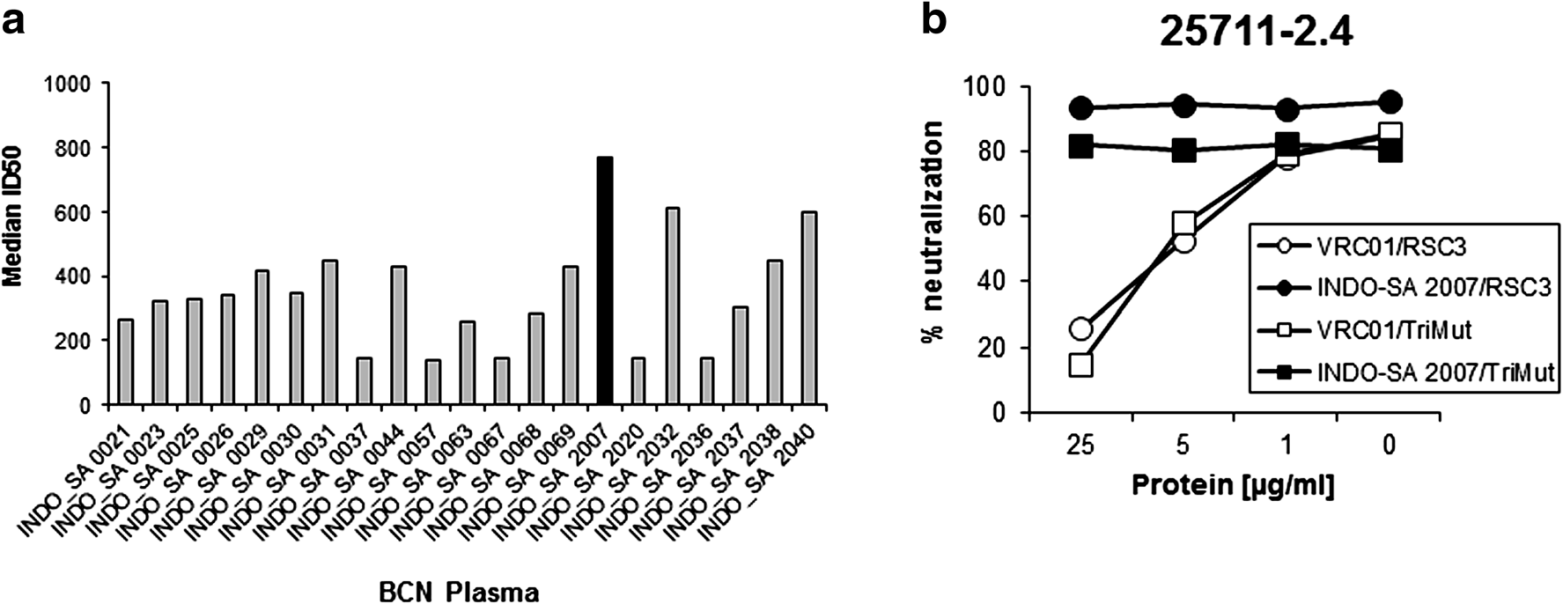
**a** Neutralization potency of BCN plasma samples against pseudoviruses expressing HIV-1 clade C Indian and South African Envs. **b** Dose–response curves showing the degree of neutralization of Env-pseudotyped virus (25711-2.4) mediated by INDO-SA 2007 plasma pre-treated with RSC3 core protein

**Table 1:** Neutralization breadth of the INDO-SA 2007 BCN plasma antibodies

Origin	Envs (n = 28)	Tier	GenBank Tier Accession No	ID_50_ values	V1 loop length	N332/N334
Negative virus controls	MuLV		DQ359272.1	<20	N/A	
	HIV-2 (7312A)		L36874	<20	N/A	
India C (n = 9)	00836-2.5	2	EF117265	450	21	Absent
	25711-2.4	1B	EF117272	2789	25	N332
	26191-2.48	2	EF117274	500	16	N332
	16055-2.3	2	EF117268	382	23	Absent
	16936-2.21	2	EF117270	694	20	N332
	4-2.J41	–	GU945316.2	705	19	N334
	5-4.J16	–	GU945326.1	1233	21	N332
	7-J.20	–	EU908223.1	1384	23	N332
	11-5.J12	–	GU945332.1	2715	18	N334
South Africa C (n = 8)	Du151.2	2	DQ411851.1	249	17	N332
	Du156.12	2	DQ411852.1	770	24	N332
	Du172.17	2	DQ411853.1	1019	29	N332
	Du422.1	2	DQ411854.1	246	26	N332
	CAP45.G3	2	DQ435682.1	710	16	N334
	CAP84.32	2	EF203963.1	1017	15	N332
	CAP88.B5	2	EF203972.1	793	28	N332
	CAP239.G3	2	EF203983.1	1090	17	N332
Others (n = 11)	JRFL	2	U63632.1	97	24	N332
	JRCSF	2	M38429.1	1527	24	N332
	PVO.4	3	AY835444.1	553	29	N332
	SC422661.8	2	AY835441.1	744	23	N332
	RHPA4259.7	2	AY835447.1	871	22	N332
	92BR020	1	AY669718.1	1015	^a^	N332
	92TH021	2	AY669775.1	322	28	N334
	93IN905	2	AY669742.1	6896	15	N332
	94UG103	2	AY669705.1	108	^a^	N332
	191727_D1.12	–	HM215267.1	<50	30	–
	IAVI C22	–	–	1940	–	–
	Geometric Mean Titer			749		

^a^The V1 loop sequence of 92BR020 and 94UG103are unavailable in the NCBI GenBank database. ID50 values refer to the reciprocal dilution that conferred 50 % neutralization of viruses in a TZM-bl assay. Assays were done in duplicates and were repeated more than three times

We next examined the specificity of the INDO-SA 2007 BCN plasma. To test whether the neutralizing antibodies preferably target epitopes on monomeric gp120 or trimeric gp140, plasma was depleted with monomeric gp120 (from strain 4-2.J41 containing N334) [29] and trimeric gp140 (made using BG505-SOSIP.664 containing N332) proteins [30]. As shown in Table 2A, we found a significant reduction in neutralization sensitivity of selected heterologous Env-pseudotyped viruses to INDO-SA 2007 BCN plasma depleted with both monomeric gp120 and trimeric gp140. We next tested if the INDO-SA 2007 bnAb possess specificity to known bnAb targets such as (1) CD4bs by examining neutralization of Env-pseudotyped viruses (25711-2.4) in the presence of the CD4bs competitors, RSC3 and TripleMut proteins [31, 32], (2) V2/V3 epitopes by examining ability of the BCN plasma antibodies to neutralize a battery of Env mutants that removes the key epitopes targeted by bnAbs and (3) MPER by examining neutralization of HIV-2/HIV-1 MPER chimeric viruses to the BCN plasma antibodies [33, 34]. The INDO-SA 2007 BCN plasma pre-treated with RSC3 and TripleMut core protein was found to show comparable neutralization of Env-pseudotyped virus (25711-2.4) to that of the plasma not treated with RSC3 core protein (Fig. 1b). Our data indicated that the INDO-SA 2007 BCN plasma did not possess CD4bs directed antibodies associated with virus neutralization. When examined for the presence of antibodies that are dependent on known epitopes in gp120 variable regions, neutralization by INDO-SA 2007 plasma was not found to depend on epitopes such as, N160, R166, and K169 in the V2 (Table 2B). However, a 2.42 and >5-fold reduction in neutralization of the two Env-pseudotyped viruses (25711-2.4 and CAP239.G3) with N301A and N332A substitutions in V3 respectively compared to their wild types were observed (Table 2B). Finally, presence of MPER directed neutralizing antibodies was examined by investigating the degree of neutralization of HIV-2/HIV-1 chimeric viruses expressing HIV-1 clade C complete MPER region (C1C), 4E10, Z13e, 10E8 overlapping epitopes (C4) and 4E10 minimal epitope (C6). As shown in Table 2C, while antibody titer against C1C was found to be very low and insignificant (ID_50_ of 63.77), no neutralization of HIV-2/HIV-1 chimera expressing C4 and C6 constructs were observed by the INDO-SA 2007 plasma antibodies. Our data indicate that the neutralization breadth mediated by the INDO-SA 2007 plasma was not due to presence of antibodies targeting MPER. Taken together, our data indicate that the neutralizing antibodies present in the INDO-SA 2007 plasma targets both linear and conformational epitopes in gp120 and which are dependent on N332 glycan in the V3 region. Nonetheless, as shown in Table 1, since the INSO-SA 2007 plasma was found to also neutralize pseudoviruses expressing Envs lacking N332 (such as 00836-2.5 and 16055-2.3), it likely has other specificities besides N301 and N332.

**Table 2:** A. Fold changes in neutralization sensitivity of the Env-pseudotyped viruses to INDO-SA 2007 plasma depleted with the monomeric (4-2.J41) gp120 and trimeric (BG505-SOSIP.664) gp140 proteins. B. Specificity of INDO-SA 2007 BCN plasma antibodies to known epitopes in variable loops. C. Presence of MPER directed neutralizing antibodies in INDO-SA 2007 plasma

A	Fold decrease in ID_50_^a^	
	Monomer (4-2.J41 gp120)	Trimer (BG505-SOSIP.664)
25711-2.4	>10.00	>20.00
4-2.J41	3.00	5.37
7.J20	7.06	>14.00
Du172.17	>7.00	7.14
CAP84.32	>7.00	>11.00
CAP239.G3	6.00	5.91

B			
HIV-1 Env mutants	Region	Neutralization titer (ID_50_)	Fold decrease in ID_50_^b^
HIV-1 25711-2.4	Wild type	1822.04	–
HIV-1 CAP239.G3	Wild type	1096.00	–
HIV-1 25711-2.4 N160A	V2	1484.35	1.22
HIV-1 CAP239.G3 N160A	V2	4184.00	0.26
HIV-1 25711-2.4 R166A	V2	1833.00	0.99
HIV-1 25711-2.4 K169E	V2	2587.29	0.70
HIV-1 25711-2.4 N301A	V3 stem	749.81	2.42
HIV-1 25711-2.4 N332A	V3 stem	357.36	5.09
HIV-1 CAP239.G3 N332A	V3 stem	204.60	5.35

C		
HIV-2/HIV-1 chimera	Region of HIV-1^c^	ID_50_
HIV-2 7312A	HIV-2 wild type	<20
HIV-2 7312A-C1C	Clade C MPER (ELLALDKWASLWNWFDITKWLWYIK)	63.77
HIV-2 7312A-C4	4E10 epitope (NWFDITKWLWYIK)	<20
HIV-2 7312A-C6	4E10 minimal (NWFDIT)	<20

^a^Fold reduction in neutralization of Env-pseudotyped viruses was obtained by comparing the neutralization titer (ID_50_ values) of panel viruses against undepleted and depleted INDO-SA 2007 plasma. ID_50_ values are reciprocal dilutions at which the undepleted and depleted plasma conferred 50 % neutralization of the Env-pseudotyped viruses in TZM-bl cells

^b^ID50 values refer to the reciprocal dilution that conferred 50 % neutralization of viruses in a TZM-bl assay. Assays were done in duplicates and were repeated more than three times. WT refers to wild type; MPER refers to membrane proximal external region in gp41

^c^HIV-1 MPER residues that are grafted in the HIV-2 are given in the parenthesis

We next examined the pseudotyped viruses expressing contemporaneous autologous env genes amplified from the INDO-SA 2007 plasma for their degree of sensitivity to the contemporaneous autologous plasma antibodies. The autologous *envs* were found to belong to HIV-1 clade C as determined by REGA HIV subtyping tool version 2 (http://www.bioafrica.net/rega-genotype/html/). The genetic properties of the autologous *env* clones are shown in Table 3 and Fig. 2a. Analysis of the complete amino acid (gp160) sequences of all the Envs (HIV-1 NLR-2007.J10, HIV-1 NLR-2007.J12, HIV-1 NLR-2007.J24, HIV-1 NLR-2007.J32 and HIV-1 NLR-2007.J48) revealed that they form a monophyletic cluster lineage with those of other Indian clade C Envs used in this study (Fig. 2a). Interestingly, four of the autologous Envs (HIV-1 NLR-2007.J10, HIV-1 NLR-2007.J12, HIV-1 NLR-2007.J24, HIV-1 NLR-2007.J32) obtained from this donor were found to possess longer V1 loop length with identical amino acid sequence (Fig. 2b) consisting of 41 amino acids compared to HIV-1 NLR-2007.J48 that was found to contain 27 amino acids in the V1 region. Envs with longer V1 loop were also found to possess more glycan residues (5N-linked glycan residues) than that of HIV-1 NLR-2007.J48 possessing shorter V1 loop length (2N-linked glycan residues) (Table 3). Nonetheless, all the Envs (irrespective of shorter or longer V1 loop lengths as well as variable glycan content) obtained from this donor were found to be resistant to the contemporaneous autologous INDO-SA 2007 plasma (Table 3). Since the INDO-SA 2007 plasma was found to possess N332-specific antibodies (Table 2B), we next examined the neutralization sensitivity of the autologous Envs to PGT121 and PGT128, which are potent mAbs that specifically targets these glycan epitopes. As shown in Table 3, all the autologous Envs were found to be resistant to both the mAbs (up to 10 µg/ml) thus showing a clear association between neutralization resistance of autologous Envs to INDO-SA 2007 plasma and both PGT121 and PGT128 mAbs. Sequence data revealed that except one (HIV-1 NLR-2007.J48 with shorter V1 loop length and containing a serine at the 332 position in V3 base), all the other autologous Envs contains an N332-glycan (Fig. 2c), suggesting that these later Envs were evolved with other features that prevented the N332glycan-specific neutralizing antibodies to access this epitope, possibly by concealing its optimal exposure due to a conformational change. Upon substituting the naturally occurring serine residue with a glycan at the 332 position (S332N), the sensitivity of the HIV-1 NLR-2007.J48 Env to autologous plasma, PGT121 and PGT128 mAbs was found to increase by 10, >80 and >100 folds respectively (Table 4). Thus, while the N332S mutation mediated neutralization resistance of HIV-1 NLR-2007.J48 Env, the other contemporaneous Envs despite naturally expressing N332 were found not to be susceptible to N332-specific neutralizing antibodies including PGT121 and PGT128, possibly due to attaining a distinct conformation that prevented accessing the N332 glycan epitope by these potent neutralizing antibodies.

**Table 3:** Genetic and neutralization properties of autologous *env* genes

*env clones*	Clade	% identity^a^	N332 genotype	PNLG V1 loop	PNLG V1V2 loop	Length V1 loop	Length V1V2 loop	Autologous^b^ neutralization (ID_50_)	IC_50_^c^ (µg/ml)	
									PGT121	PGT128
HIV-1 NLR-2007.J10	C	99.1	N332	5	10	41	96	36	>10	>10
HIV-1 NLR-2007.J12	C	100.0	N332	5	10	41	96	43	>10	>10
HIV-1 NLR-2007.J24	C	98.3	N332	5	10	41	96	83	>10	>10
HIV-1 NLR-2007.J32	C	99.5	N332	5	10	41	96	56	>10	>10
HIV-1 NLR-2007.J48	C	95.0	S332	2	07	27	76	25	>10	>10

^a^ % identity of the autologous env amino acid sequences to NLR-2007.J12 was deduced using LAALIGN tool (http://www.ch.embnet.org/software/LALIGN_form.html). It refers to the degree of correlation between two un-gapped sequences and indicates that the amino acid at the particular position is an exact match

^b^ID50 values refers to the reciprocal dilution of the plasma that mediated 50 % neutralization of the HIV-1 Env pseudotyped viruses in TZM-bl cell

^c^IC50 values indicate concentration that mediated 50 % Env-pseudotyped virus neutralization in TZM-bl cells. Note that in our study, the highest mAb concentration used was 10 µg/ml

**Fig. 2:**
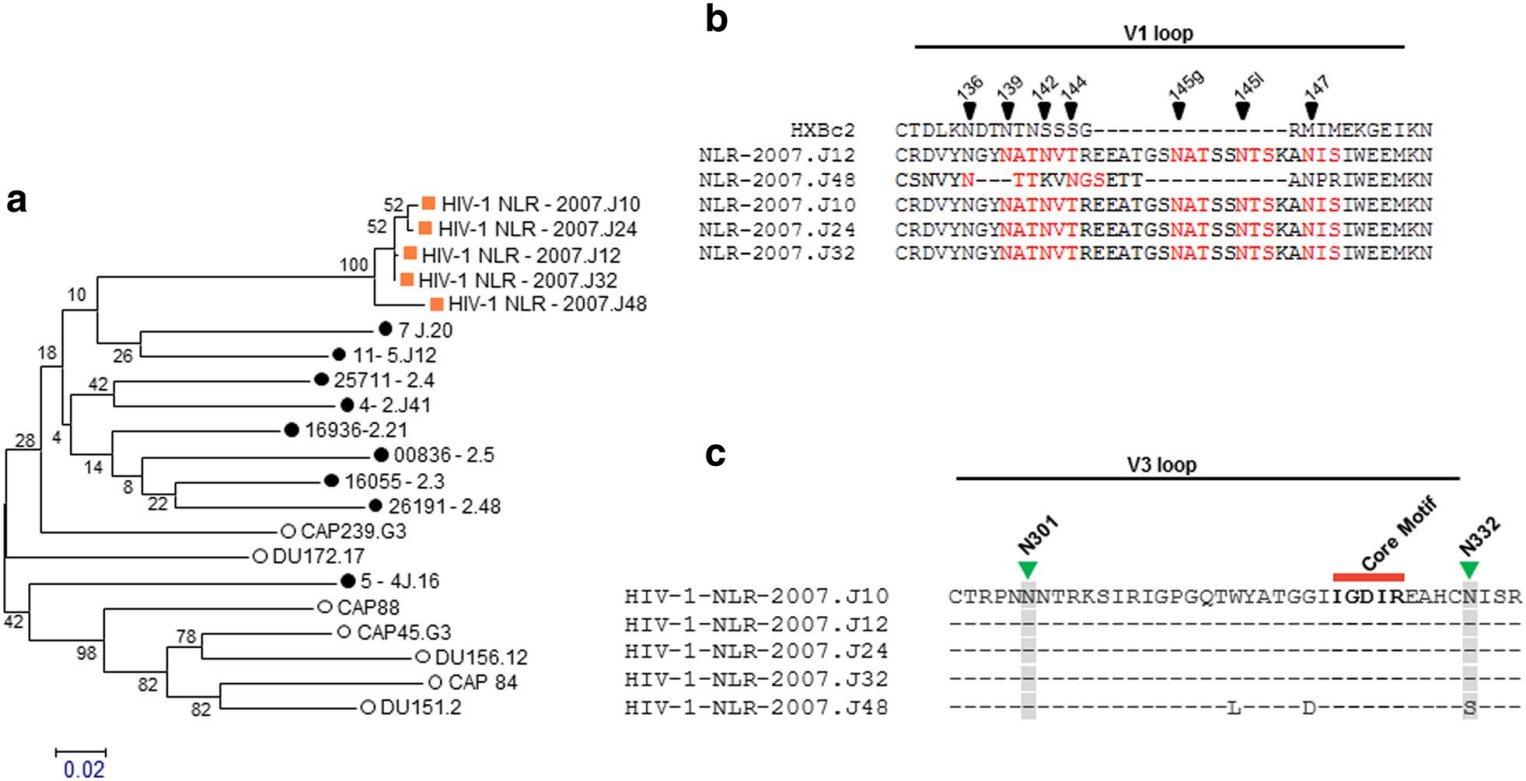
**a** Inter and intra-clade genetic relatedness of INSO-SA 2007 autologous HIV-1 clade C *env (gp160)* amino acids. Maximum likelihood phylogenetic tree was constructed with 50 bootstrapped replicates and inferred by using Jones–Taylor–Thornton (JTT) model [35]. Values next to the nodes of each branch indicate percentage of trees in which the associated taxa are clustered together. Branch length represents residues substitution per site. The scale shown at the bottom of the tree indicates 0.02 substitutions per amino acid residue. **b** Alignment of amino acid sequences of V1 loop of autologous *envs*. N-linked glycans are highlighted in *red* and their positions are highlighted. **c** Comparison of amino acid sequences between the V3 region of the autologous HIV-1 *env* clones. Amino acid sequences were aligned using ClustalW algorithm using Mega 5.2 program and the alignment file was further processed in *Seqpublish* tool which is available in www.hiv.lanl.gov. The N301 and N332 glycan residues including the core amino acid motif IGDIR important for PGT128 sensitivity are highlighted. Amino acid numbering was done relative to HXBc2 (http://www.hiv.lanl.gov/content/sequence/HIV/REVIEWS/HXB2.html)

**Table 4:** Association of V1 loop length and its glycan content with sensitivity of Env-pseudotyped viruses to neutralizing mAbs

Envelopes and Chimera	INDO-SA 2007 BCN plasma (ID_50_ values)	IC_50_ (µg/ml)					
		b12	VRC01	PG9	PG16	PGT121	PGT128
HIV-1 NLR 2007.J12	43	>10	>10	0.04	0.14	>10	>10
HIV-1 NLR 2007.J48	25	>10	>10	0.06	0.08	>10	>10
HIV-1 NLR 2007.J48 (S332N)	250	>10	>10	0.09	0.07	0.12	0.08
HIV-1 NLR 2007.J48 (V1) in HIV-1 NLR 2007.J12	208	>10	>10	0.22	<0.04	0.29	0.13
HIV-1 NLR 2007.J12 (V1) in HIV-1 NLR 2007.J48 (S332 N)	96	>10	>10	0.33	0.66	2.14	>10
HIV-1 NLR 2007.J12 (Δ V1 N-glycans)	84.88	>10	>10	0.09	2.56	0.46	0.27

To further elucidate the cause of resistance of N332-glycan containing autologous Envs to N332-glycan specific INDO-SA 2007 bnAb plasma activity, PGT121 and PGT128 mAbs, we first compared the amino acid sequences of the HIV-1 NLR-2007.J48 with that of other four contemporaneous Envs (HIV-1 NLR-2007.J10, HIV-1 NLR-2007.J12, HIV-1 NLR-2007.J24 and HIV-1 NLR-2007.J32) that naturally contain the N332 glycan residue. All these Envs were found to have identical V3 loop sequence (except for HIV-1 NLR-2007.J48 having two amino acid differences) and contained the IGDIR motif including the glycans in the V3 loop shown to be important for PGT128 sensitivity [28]. Interestingly, we found a positive association between shorter V1 loops and susceptibility of the heterologous Envs tested against the INSO-SA 2007 bnAb plasma activity (Table 1) too. In order to determine whether longer V1 loop length was associated with neutralization resistance of autologous Envs to N332 directed neutralizing antibodies, we next exchanged the V1 domain between HIV-1 NLR-2007.J48 (S332N) and HIV-1 NLR-2007.J12 and examined susceptibility to the INDO-SA 2007 plasma, PGT121 and PGT128 mAbs. As shown in Table 4, while HIV-1 NLR-2007.J12 expressing V1 region of HIV-1 NLR-2007.J48 (S332N) (shorter V1 loop) became sensitive to both PGT121 and PGT128 mAbs; HIV-1 NLR-2007.J48 (S332N) containing V1 loop of the HIV-1 NLR-2007.J12 Env became resistant to the PGT121 and PGT128 mAbs by >17 and >100-fold respectively. As shown in Table 3 and Fig. 2b, we also found that while HIV-1 NLR-2007.J48 contains two glycan residues (N136, N144), the other four contemporaneous Envs contain five glycan residues (N139, N142, N145g, N145l, N147) within the V1 loop. Interestingly, as shown in Table 4, presence of N136, N144 residues in the HIV-1 NLR-2007.J48 did not modulate its sensitivity to the neutralization by both autologous BCN plasma and the PGT121 as well as PGT128 mAbs, suggesting that these two glycans did not play any protective role. So, to further examine whether glycan residues present in the other Envs with longer V1 loop length as shown in Table 3 played any role in neutralization resistance to the antibodies tested in our study, we carried out site-directed mutagenesis to substitute N-glycans with alanine residues. As shown in Table 4, knocking out of these glycan residues was found to be correlated significantly with increased susceptibility of the NLR-2007.J12 Env (N139A/N142A/N145gA, N145lA/N147A) with longer V1 loop to PGT121 and PGT128 mAbs by >20 and >35 folds respectively. However, only 1.9-fold increase in neutralization of NLR-2007.J12 (N139A/N142A/N145gA, N145lA/N147A) to autologous plasma antibodies was observed. Our data indicated that while incorporation of protective glycan residues conferred significant resistant of Envs to PGT121 and PGT128 mAbs, it moderately mediated resistance of Envs to contemporaneous INDO-SA 2007 plasma antibodies, indicating that longer V1 loop length had more influence on neutralization resistance over the protective glycan residues. Interestingly, as per the HIV Los Alamos CATNAP database (http://www.hiv.lanl.gov/content/immunology/neutralizing_ab_resources.html), viruses containing N332 glycan residue and with unusually longer V1 loop length (37–47 amino acids) were found to be resistant to PGT121 (67 %) and PGT128 (59 %) mAbs, indicating a modest association between V1 loop length and virus resistance to PGT121/128 mAbs. Our study also demonstrated that neither longer V1 loop length nor its glycosylation pattern was found to influence the susceptibility of Env to b12, VRC01 mAbs, which is in contrast to the recent finding by van den Kerkhof et al. [14] where longer V1 loop length associated with resistance to b12 mAb was reported.

In summary, our data provide evidence that the circulating HIV-1 clade C in this elite neutralizer escaped the neutralization by the autologous plasma in this patient via three distinct mechanisms: (1) due to a N332S mutation (2) by increasing V1 loop length and (3) incorporation of protective N-glycan residues in V1 loop. These features hindered the neutralizing antibodies, developed in this donor, to optimally access the N332 epitope. Additionally, we show that these features also conferred resistance to PGT121 and PGT128 mAbs that also targets N332 epitope in the V3 base. Although an association between expanded V1 loop length and sensitivity of HIV-1 Env to PGT135 mAb but not to the PGT121 and PGT128 mAbs have very recently been demonstrated [14], our observation on the association between expanded V1 loop with resistance to PGT121 and PGT128 was possibly due to differences in the angle of approach of these mAbs to N332 glycan residue compared to that of PGT135 as demonstrated by van den Kerkhof et al. [14] and Kong et al. [25].
